# Assessing the Diagnostic Performance of Automated Pituitary Gland Volume Measurement for Idiopathic Central Precocious Puberty

**DOI:** 10.3390/jcm14010015

**Published:** 2024-12-24

**Authors:** Hayoun Kim, Inkyu Yu

**Affiliations:** Departments of Radiology, Eulji University Hospital, Eulji University College of Medicine, 95 Dunsanseo-ro, Seo-gu, Daejeon 35233, Republic of Korea

**Keywords:** precocious puberty, pituitary gland volume, multi-scale attention network (MANet)

## Abstract

**Background/Objectives:** It is known that the pituitary gland volume (PV) in idiopathic central precocious puberty (IPP) is significantly higher than in healthy children. However, most PV measurements rely on manual quantitative methods, which are time-consuming and labor-intensive. This study aimed to automatically measure the PV of patients with IPP using artificial intelligence to accurately quantify the correlation between IPP and PV, and to improve the efficiency of diagnosing IPP. **Methods:** From July 2016 to February 2024, 226 patients who had been diagnosed with IPP and undergone brain MR imaging were included (117 males and 109 females; median age, 8 years; interquartile range, 7–9 years). A control group of 52 patients who had undergone brain MR imaging without symptoms of precocious puberty was also included (37 males and 15 females; median age, 8 years; interquartile range, 8–9 years). Measurement variability was examined between manual and automatic measurements (n = 57). The pituitary gland volume was measured using 1–3 mm thickness T1 sagittal images from non-enhanced brain MR imaging, analyzed with the MA-net artificial intelligence learning method. Physical characteristics (height, weight, and age) were correlated with PV, and the difference in PV between the IPP group and the control group was evaluated. **Results:** The intraclass correlation coefficient was 0.993 for agreement between manual and automatic measurement. Confounding bias was reduced by PSM. PV was positively correlated with age and body weight in the IPP group (17.4%, *p* = 0.009, and 14.0%, *p* = 0.037). The median values of PV were 432 mm³ in the IPP group and 380 mm³ in the control group, showing a significant difference of 52 mm³ (*p* < 0.05). **Conclusions:** The PV in the IPP group was significantly higher than in the control group. Automatically measuring PV along with assessing hormone levels could enable a faster and more straightforward diagnosis of IPP.

## 1. Introduction

Precocious puberty is characterized by the early onset of secondary sexual characteristics, occurring more than two standard deviations earlier than the average, with breast development before age 8 in girls and testicular development before age 9 in boys [1]. Recent trends indicate an earlier onset of puberty globally, as reported by the Pediatric Research in Office Setting (PROS) study of 17,000 healthy girls in the U.S., which showed earlier breast and pubic hair development [2].

Precocious puberty is classified into true (central, gonadotropin-dependent) and pseudoprecocious (gonadotropin-independent). True precocious puberty results from early maturation of the hypothalamus–pituitary–gonadal axis, while pseudoprecocious puberty arises from excessive sex hormone secretion by the gonads or adrenal glands. True precocious puberty occurs in 1 in 5000–10,000 people, often sporadically, and is 10 times more common in girls than in boys. In girls, 80–90% of cases are idiopathic, while central nervous system abnormalities are found in 25–50% of cases in boys [1,3].

Early onset of secondary sexual characteristics and rapid bone maturation may be accompanied by decreased final adult height, psychosocial problems, and behavioral problems. Since the causes include diseases that require early detection, such as central nervous system tumors or ovarian tumors, accurate diagnosis and treatment are required [3]. The gonadotropin-releasing hormone (GnRH) stimulation test is the standard diagnostic method for assessing hypothalamic–pituitary–gonadal activation, but it has limitations, including low sensitivity, high cost, invasiveness, and long duration. Thus, there is a need for a non-invasive diagnostic approach [4].

MRI is preferred for evaluating the pituitary gland to rule out brain abnormalities in true precocious puberty. When evaluating the pituitary gland on MRI, it has been reported that true precocious puberty is associated with a greater height of the pituitary gland compared to the normal group [5]. During puberty, the pituitary gland grows, generally reaching 9 mm in height in girls and 8 mm in boys. Previous study showed that higher pituitary gland stages or heights on MRI correlate with a higher predictive value for diagnosing true precocious puberty, though sensitivity remains low [5].

Moreover, recent studies showed that PV (pituitary gland volume) is significantly larger in patients with IPP (idiopathic central precocious puberty) compared to normal individuals. This suggests that measuring pituitary volume could be useful for diagnosing idiopathic precocious puberty. According to Su Wu et al., in a study involving 90 patients with premature breast development, 133 patients with IPP, 35 patients with early puberty, and 30 normal subjects, PV was identified as a predictive indicator for diagnosing IPP. The study reported a sensitivity of 54.10%, a specificity of 72.20%, and a cutoff value of 196.01 mm³ [1]. PV was significantly correlated with several hormones [1,6]. It was also reported that bone age could be used as a potential indicator for diagnosing IPP [1,3].

There are several methods to measure pituitary volume. It can be evaluated qualitatively by grading based on its shape, and this method is subjective. Another approach involves estimating the volume by measuring the height of the pituitary gland [7,8]. Pituitary volume can also be quantitatively assessed using mathematical techniques such as the elliptic formula, planimetry, and point counting. While manual quantitative analysis, where measurements are taken directly from MRI images, offers objective evaluation, it is limited by the considerable time required for repetitive tasks, which affects efficiency.

In contrast, automatic quantitative analysis using artificial intelligence offers a more efficient and objective way to measure pituitary volume, reducing time and effort. Although small size and shape variation of PV is a specific challenge, recent advancements in deep learning, especially convolutional neural networks (CNNs), demonstrated promising results in medical image segmentation [9,10,11].

The aim of this study was to determine whether there was a difference in PV obtained using a multi-scale attention network (MANet)-based analysis method from MRI between an IPP group and a control group. In addition, this study analyzed the correlation between the automatically measured PV and factors such as height, weight, and age in patients within the IPP group.

## 2. Materials and Methods

### 2.1. Subjects

Institutional Review Board approval was obtained (IRB No. EMC 2023-10-008). From July 2016 to February 2024, among patients who visited the hospital with symptoms suspected of precocious puberty, those diagnosed with central precocious puberty through the GnRH stimulation test were included. A number of 226 patients who had been diagnosed with true precocious puberty and had undergone brain MRI and 52 control subjects who had visited the hospital with symptoms such as headaches but no symptoms of precocious puberty and had undergone brain MRI were included in the study. Those with pituitary lesions were excluded from the study. Subjects with end-organ dysfunction, which is considered a factor increasing PV, were aslo excluded. The height, weight, and age of the patients were recorded.

### 2.2. Neuroimaging

MRI was performed on 3T (3T Skyra, Magnetom Lumina, Siemens Healthcare, Erlangen, Germany) machines. T1 sagittal images cut at a section thickness of 1 mm (TR/TE: 1690/3.1, matrix: 352 × 246, FOV:180, flip angle: 150, section thickness: 1 mm, section number: (1)) and 3 mm (TR/TE: 271 × 2.9, matrix:512 × 264, FOV: 2 mm, flip angle: 75, section thickness: 3 mm, section number: (2)) were remodeled to create three-dimensional images (Table 1).

### 2.3. GnRH Stimulation Test

After slowly injecting Relefact LH-RH 100 ug intravenously or decapeptyl 0.1 mg subcutaneously, LH and FSH values were measured at 0, 30, 60, and 90 min after. If the peak LH level in the GnRH stimulation test was 5 mIU/mL or higher, it was diagnosed as true precocious puberty; if it was less than 5 mIU/mL, it was diagnosed as pseudoprecocious puberty. True precocious puberty was classified into central precocious puberty, where abnormalities in the central nervous system were detected through pituitary MRI, and idiopathic true precocious puberty, where no identifiable cause was found.

### 2.4. Manual PV Measurement

The voxel counting method within a region of interest was used for PV measurement. One radiologist and one neuroradiologist manually annotated the pituitary gland on each sagittal section to measure its volume. In cases where the two radiologists had a different opinion, the boundaries of the pituitary gland were determined through consensus.

### 2.5. Automated PV Measurement

#### 2.5.1. Preprocessing

**Figure 1:**
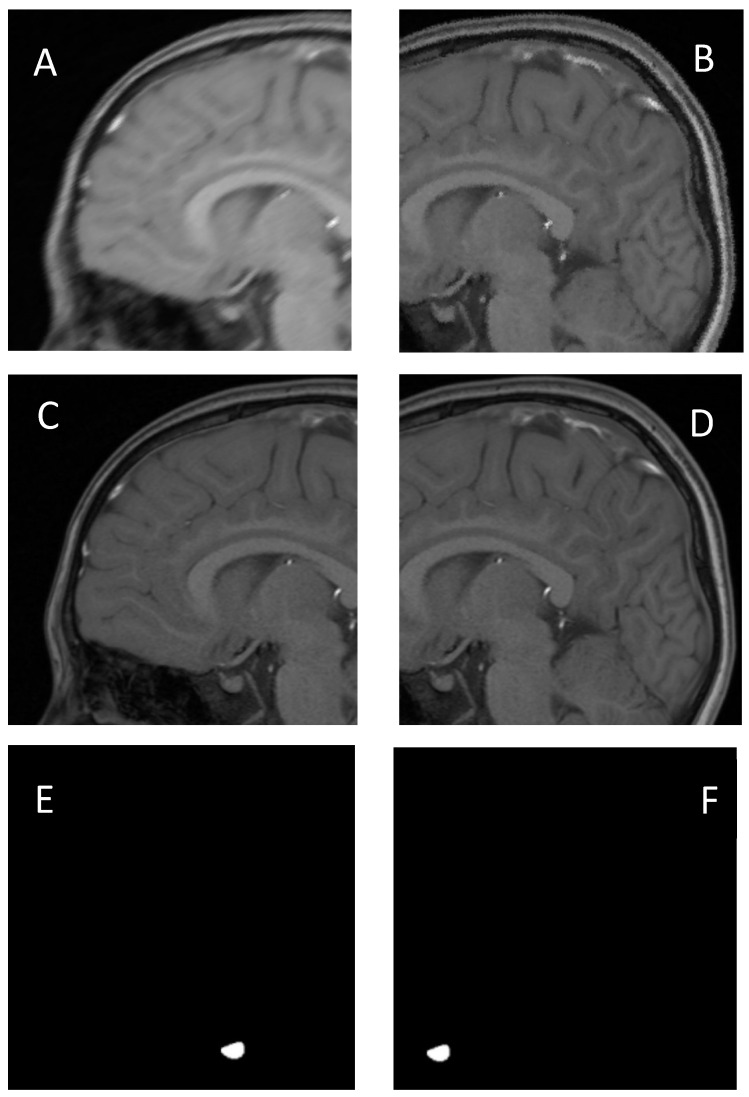
The preprocessing steps. (**A**,**B**), Patched image from the resized image. (**C**,**D**), Augmented image from the patched image. (**E**,**F**), Mask image.

Due to the small size of the pituitary gland, an MRI scan of a patient typically provided around 15 slices; therefore, there was insufficient training data. The preprocessing steps included patching and data augmentation techniques such as blurring and adding noise to enhance the robustness and generalizability of the model. To prepare an image for patching, the original image (320 × 320) was resized (384 × 384). Using the resized image (384 × 384 × 1), overlapping patches (256 × 256 × 4) were created, followed by data augmentation (Figure 1).

#### 2.5.2. MANet Architecture

**Figure 2:**
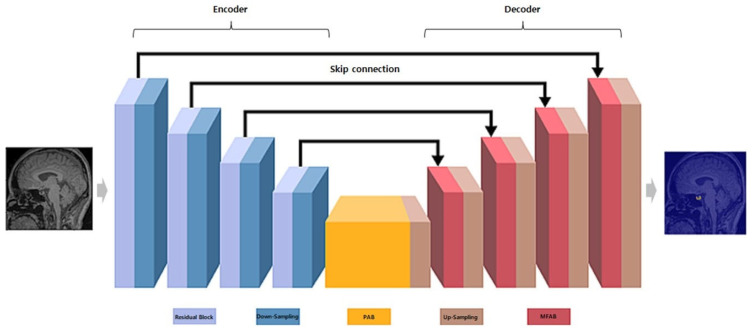
MANet architecture. Encoder utilizes a series of convolutional layers with varying kernel sizes to capture features at multiple scales, followed by pooling layers for down-sampling and reducing spatial dimensions [12]. Decoder employs up-sampling layers and skip connections from the encoder to reconstruct the segmentation map, preserving spatial information and enabling precise edge detection [12]. Attention block implements spatial and channel-wise attention mechanisms to emphasize relevant features and suppress irrelevant ones, thereby enhancing the capture of characteristics related to the pituitary gland. The position-wise attention block (PAB) captures spatial dependencies between pixels in feature maps and focuses on spatial attention mechanisms [13]. The multi-scale fusion attention block (MFAB) captures channel dependencies across feature maps at multiple levels and applies channel-wise attention mechanisms [13].

MANet, a variant of U-Net, improved segmentation by using multi-scale fusion attention blocks (MFAB) to emphasize key features at various encoding and decoding levels, rather than simply aggregating them as in U-Net (Figure 2).

The network consisted of the following key components:

- Encoder: Utilizes a series of convolutional layers with varying kernel sizes to capture features at multiple scales, followed by pooling layers for down-sampling and reducing spatial dimensions [12].
- Decoder: Employs up-sampling layers and skip connections from the encoder to reconstruct the segmentation map, preserving spatial information and enabling precise edge detection [12].
- Attention block: Implements spatial and channel-wise attention mechanisms to emphasize relevant features and suppress irrelevant ones, thereby enhancing the capture of characteristics related to the pituitary gland.

The position-wise attention block (PAB) captures spatial dependencies between pixels in feature maps and focuses on spatial attention mechanisms [12,13]. The multi-scale fusion attention block (MFAB) captures channel dependencies across feature maps at multiple levels and applies channel-wise attention mechanisms [13,14].

#### 2.5.3. Training

The network was trained using a combination of Tversky loss and Jaccard loss, both based on the Dice coefficient, to optimize pixel-level accuracy and ensure robust performance in various aspects of the segmentation task. The Adam optimizer was employed with an initial learning rate of 0.001. Training was conducted for 100 epochs with a batch size of 64, using a Tesla V100.

#### 2.5.4. Inference

**Figure 3:**
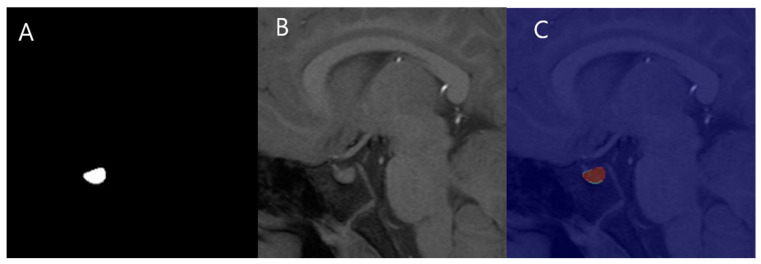
Interference. (**A**), Cropped input image. (**B**), Mask image. (**C**), Overlapped image.

The outer regions are cropped, and only a 256 × 256 central crop is used as input as in Figure 3. During output, zero-padding is applied to the cropped areas to maintain the original image dimensions.

### 2.6. Statistics

To assess the variability of PV measurements, the agreement between manual and automated quantitative analysis was evaluated using the intraclass correlation coefficient (ICC). Depending on the characteristics of the collected variables, categorical variables were analyzed using frequency and chi-square tests, while continuous variables were analyzed using the Mann–Whitney test after a normality test. The correlation between height, weight, age, and PV in the IPP patient group was analyzed. The difference in PV values between the IPP group and the control group was determined. The analysis software used was IBM SPSS/WIN ver 25.0 (IBM Corp., Armonk, NY, USA), and the statistical significance level was set at *p* < 0.05.

## 3. Results

A number of 226 patients diagnosed with IPP were included (117 males and 109 females, mean age: 8.12, range from ages 4 to 9), and 52 patients were set as the control group (37 males and 15 females, mean age: 7.79, range from ages 4 to 9). Physical characteristics (sex, age, height, and weight) of the two groups are shown in Table 2.

**Figure 4:**
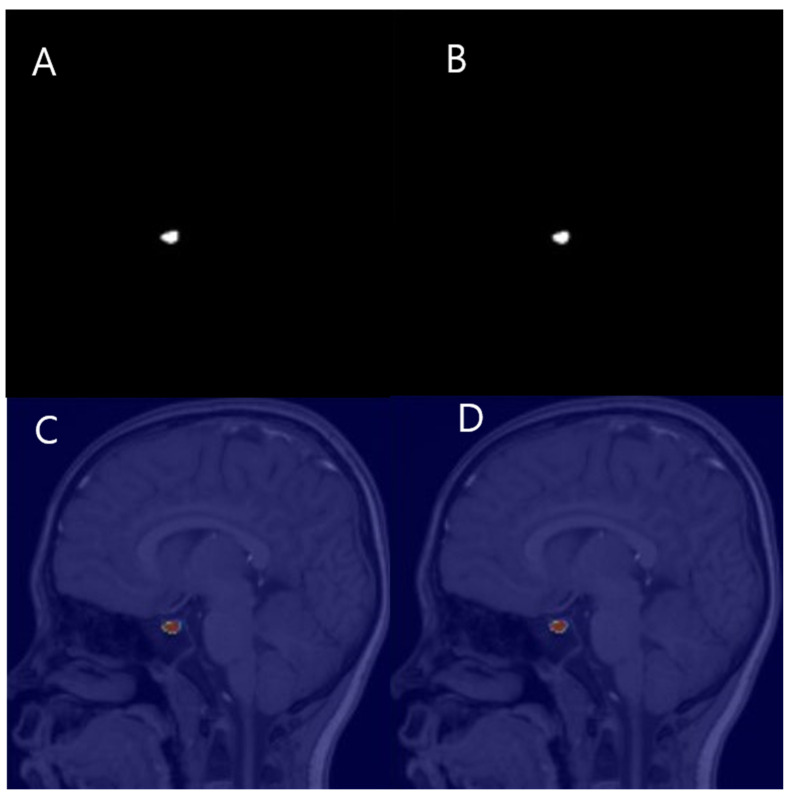
A patient with 99.56% agreement between manual and automated PV measurement. (**A**,**B**), Mask image of manual (**A**) and automated (**B**) measurement. (**C**,**D**), Overlapped image of manual (**C**) and automated measurement (**D**).

Measurement variability was examined between manual and automatic measurements (n = 57). The intraclass correlation coefficient was 0.993 for agreement between manual and automatic measurement (Table 3, Figure 4).

Confounding bias was reduced by propensity score matching (PSM). In the IPP group, the correlation between age and PV was 17.4%, and the *p*-value was 0.009, which was statistically significant. The *p*-value for relation of PV with weight came out to be 0.037 in the IPP group which was statistically significant, and the correlation was 14.0%. In the IPP group, the correlation between height and PV was not statistically significant. In the control group, the correlation between age and PV was 34.7%, and the *p*-value was 0.012, which was statistically significant. A *p*-value for the relation of PV with height came out to be 0.001 in the control group which was statistically significant, and the correlation was 43.9%. In the control group, the correlation between body weight and PV was 24%, but it was not statistically significant (Table 4).

The median PV in the IPP group came out to be 432 mm^3^ (interquartile range, 352–493 mm^3^). In the control group, the median PV was 380 mm^3^ (interquartile range 301–460 mm^3^) (Table 5). The difference in PV between the IPP group and the control group was 52 mm^3^, which was statistically significant.

## 4. Discussion

Since PV is highly correlated with precocious puberty [1,5], measuring PV using MRI can predict the diagnosis and serves as a non-invasive test. Pituitary volume has been measured using various methods (Table 6). First, the evaluation of pituitary shape is performed according to the outline of the surface above the pituitary gland in the midline plane, and can be divided into five stages: stage 1 = distinct concave, stage 2 = slightly concave, stage 3 = flat, stage 4 = slightly convex, and stage 5 = marked convex. It has the advantage of being simple to evaluate by grading the height of the pituitary gland observed with the naked eye, but has the limitation of being a subjective method [5]. Second, there is also a method to estimate the volume by measuring the height of the pituitary gland, and the longest vertical distance between the base and the apex of the pituitary gland is measured on the midline sagittal plane of the T1-weighted image [7,8]. This method is simple and provides a numerical value, but it may not be accurate enough to reflect the total PV.

PV can be also quantitatively evaluated using mathematical approaches. First, there is a method using the elliptic formula. The maximum height and maximum length are measured in the mid-sagittal plane of the T1-weighted image, and the maximum width is measured in the coronal plane. Then, the PV is estimated by considering the maximum variability of the pituitary shape using the height × length × width/2 formula [1,15,16,17]. This measurement is valid in ellipsoid shaped glands and has the limitation that it is an estimated value.

The second is the planimetry method, which manually draws the entire pituitary volume using the Visage 3D polygonal region of interest (ROI) tool. The ROI is manually drawn layer by layer using the mouse guided cursor. The border of the pituitary gland is defined by the sphenoid sinus below and the diaphragma sella above, and is performed for all sagittal slices. The PV is calculated using the ROI and section thickness of all layers [18,19,20]. The third is a method obtained through point counting. The above mentioned manual quantitative analysis, in which a person directly measures and calculates the size through MRI images, is an objective evaluation method, but it has limitations in efficient use due to the large amount of time consumed by repetitive simple tasks. Therefore, automated methods for measuring pituitary volume are a way to save time and effort.

According to a previous study, the average PV value was 200.17 ± 67.33 mm^3^ in girls with IPP and 200 ± 0.7 mm^3^ in all boys and girls aged 1–10 years [21] when the PV was calculated using the elliptical formula. In our study, the median PV value of the IPP group showed 432 mm^3^, and that of the control group showed 380 mm^3^. This is higher than the values reported in previous studies. In another previous study [22], PV was calculated using the elliptical formula and the voxel values calculated by drawing an ROI in a group corresponding to the adolescent age group were compared. As a result, the mean PV using the voxel counting method was 0.54 cc ± 0.16 cc, and the mean PV using the elliptical formula was 0.42 cc ± 0.16 cc, and the two methods showed a significant positive correlation. When measuring PV using the ellipsoid formula, it was reported that the values obtained were lower compared to those measured using voxel counting or planimetry [22,23]. This result can explain the discrepancy between the PV values in this study and those reported in previous literature. In another study in 2021, when the pituitary volume value was obtained using the voxel counting method, the average value was 378.8 ± 65.8 for boys and 436.7 ± 165.4 for girls, showing similar values to those in this study [24].

Previous studies showed that PV values were significantly larger in females in the same age group of obese patients [19], in the adolescent age group and in the 0–18 age group [16]. The PV value was larger in boys from 0 to 6 years of age, and larger in girls from 6 to 12 years of age [24]. In this study, there was no significant difference in pituitary volume values by gender.

Many previous studies have reported that the value of PV increases with age during prepubertal years and peaks in the early 20s [1,17,21,22,24]. Three studies emphasized that PV reaches its maximum size in early adulthood and that its decline later in life is a consistent pattern observed in both genders [17,20,21]. In this study, there was a significant correlation with age with a *p* value of 0.009 in the IPP group (17.4%) and a *p* value of 0.012 in the control group (34.7%), which is consistent with previous results. This study, which focused on children, confirmed that the trends and results observed are consistent with the expected physiological changes during growth.

A moderate correlation was observed between pituitary volume (PV), height, body weight, and age in the control group in this study. The correlation between pituitary volume (PV) and factors such as height, weight, and age in this study is likely attributed to physiological changes during the growth process. As the body matures, these factors are interconnected, with the pituitary gland playing a significant role in regulating growth and development. In children diagnosed with idiopathic central precocious puberty (IPP), this correlation might be more pronounced due to the early onset of puberty, which can lead to changes in growth patterns and hormonal regulation. Further studies are needed to explore these relationships in greater depth and to clarify how growth-related physiological changes influence the pituitary gland’s volume and its correlation with height, weight, and age.

In our study, it was observed that children in the IPP group were taller than those in the control group. Idiopathic central precocious puberty (IPP) is associated with early growth acceleration, which can result in a temporary increase in height compared to peers in the control group. This early growth spurt, however, is often followed by a reduction in final adult height due to premature closure of the epiphyseal growth plates [25].

In this study, to evaluate the accuracy of the automatic PV measurement program, we performed an agreement analysis comparing the values measured manually and the values measured by AI, which showed very high agreement (ICC = 0.993). The automated measurement technique using MANet, (version 0.3.3, XCUBE.Co., Ltd., Daegu, Republic of Korea) ensures high accuracy and reproducibility, which is believed to minimize the subjective judgment of researchers.

One of the key advantages of using automated methods for PV measurement, as demonstrated in previous studies, is the ability to achieve higher accuracy and reproducibility. For example, automated measurements have shown a high intraclass correlation coefficient (0.993) with manual measurements in our study, which underscores their reliability in clinical applications. This level of consistency is crucial when assessing PV as a potential marker for IPP, where precision is vital for accurate diagnosis and monitoring [1].

Additionally, automated techniques offer significant improvements in efficiency compared to manual methods, which are often time-consuming and labor-intensive. This efficiency allows clinicians to process larger datasets more quickly, potentially enabling faster diagnoses and more timely interventions, which are essential for managing conditions like IPP. Early identification of IPP is important because it facilitates the timely administration of treatments that can mitigate associated risks such as abnormal growth patterns and psychosocial issues [3].

Furthermore, the integration of artificial intelligence (AI) in PV measurement improves diagnostic accuracy by eliminating human error and standardizing the process. AI algorithms can analyze MRI scans objectively, providing consistent measurements that are less prone to variability than manual methods. This consistency is particularly beneficial in clinical settings, where imaging conditions may vary, and helps reduce diagnostic ambiguity [26].

Limitations of the current work include small sample size study. The small sample size in the control group may limit the statistical power of the study, a limitation due to constrained resources and data accessibility. Second, there was a lack of comparison with factors such as BMI, bone age, and hormone levels. Additional investigations into these factors are needed for a more detailed analysis. If there is research that allows artificial intelligence to detect subtle changes or abnormal findings in PV, it will be helpful in more accurately and specifically identifying the relationship with diseases such as endocrine disorders. AI-based methods for measuring pituitary volume (PV) show significant clinical potential, but their adoption faces challenges in generalizability, interpretability, validation, and integration. Variability in MRI equipment and protocols can limit generalizability, while the opaque nature of deep learning models impacting interpretability and clinician trust. Rigorous validation through expert comparisons, clinical trials, and periodic revalidation is essential to ensure accuracy and reliability. For seamless integration, AI systems must be compatible with electronic health records (EHR), provide clear and actionable outputs, and include clinician training. Addressing these challenges through research, audits, and feedback will enable effective implementation and equitable patient outcomes.

## 5. Conclusions

The PV of in the IPP group was measured to be significantly higher than that measured in the control group. Considering automatically measured PV as well as hormone levels may help to give a quick and simple diagnosis of IPP.

## Figures and Tables

**Table 1:** MRI protocol used in the present study.

	Precontrast T1 Sagittal Image	
SL, mm	1	3
TR, ms	1690	271
TE, ms	3.1	2.9
Matrix	352 × 246	512 × 264
FOV, mm	180	2
Section number	1	2
Flip angle	150	75

FOV = field of view, SL = slice thickness, TE = echo time, T1 = inversion time, TR = repetition time.

**Table 2:** Patient IPP for demographic characteristics.

	Control	IPP	Total
Number of patients	52	226	278
Sex (female)	15/52	109/226	124/278
Age, year (range) ^a^	8 (7–9)	8 (8–9)	8 (8–9)
Height (cm) ^a,^*	124 (119–136)	138 (132–142)	136 (128–141)
Weight (kg) ^a,^*	27 (22–37)	35 (29–45)	35 (27–44)

Note: IPP, patients diagnosed with idiopathic central precocious puberty and had undergone brain MRI; control, patients who had undergone brain MRI without symptoms of precocious puberty. ^a^ Numbers in parentheses are interquartile ranges. * *p*-values at a significance level of 0.05.

**Table 3:** Intra-class correlation coefficient.

	ICC	CI 95%		Actual Value 0 for F-test			
		LLCI	HLCI	F	df1	df2	*p*-Value
Single measure	0.993	0.988	0.996	285.787	56	56	<0.001 *
Average measure	0.997	0.994	0.998	285.787	56	56	<0.001 *

CI; confidence interval, LLCI; lower limit of the confidence interval, HLCI; higher limit of the confidence interval, df; degrees of freedom. * ICC agreement was significant at *p* < 0.05.

**Table 4:** Correlation between pituitary volume and demography in IPP patients.

	Control	IPP
	Pituitary Gland Volume	
Height	0.439 (0.001) **	0.005 (0.93)
Weight	0.251 (0.07)	0.140 (0.03) *
Age	0.347 (0.01) *	0.174 (0.009) **

Note: Pearson correlation (* *p* values at a significance level of 0.05 * and 0.01 **).

**Table 5:** Patient IPP for pituitary gland volume.

	Control	IPP	Total
PV ^a,^*	380 (301–460)	432 (352–493)	427 (347–488)

Note: IPP, patients diagnosed with idiopathic central precocious puberty and had undergone brain MRI; control, Patients who had undergone brain MRI without symptoms of precocious puberty; PV, pituitary gland volume. ^a^ Numbers in parentheses are interquartile ranges. * *p* values at a significance level of 0.05.

**Table 6:** Methods for measuring pituitary volume.

Method	Description	Advantages	Limitations
Evaluation of Pituitary Shape	Assesses pituitary shape based on the surface outline in the midline plane, divided into 5 stages: 1. Distinct concave; 2. Slightly concave; 3. Flat; 4. Slightly convex; 5. Marked convex	Simple and quick to evaluate by visual grading	Subjective method with potential for observer bias
Height Measurement Method	Measures the longest vertical distance between the base and apex of the pituitary gland in the midline sagittal plane using T1-weighted images	Simple to perform; provides numerical values	May not accurately reflect total PV
Elliptic Formula Method	Measures the maximum height and length in the mid-sagittal plane and maximum width in the coronal plane. PV is calculated as follows: height × length × width/2	Effective for ellipsoid-shaped glands	Provides an estimated value; may not account for all shape variability
Planimetry Method	Uses a 3D polygonal ROI tool to manually draw the pituitary’s entire volume, layer by layer, on sagittal slices. PV is calculated using the ROI and section thickness	Accurate and considers entire gland structure	Time-consuming and requires manual effort
Point Counting Method	Quantifies pituitary volume through manual measurement and point counting on MRI images	Objective and numerical	Labor-intensive and repetitive; not efficient for large datasets
Automated Measurement Methods	Utilizes automated techniques to calculate pituitary volume from imaging	Saves time and effort; reduces manual workload	May require advanced software and expertise to implement effectively

Note: PV, pituitary gland volume; ROI, region of interest.

## Data Availability

The data supporting the reported results cannot be shared due to privacy or ethical restrictions.

## Abbreviations

GnRH	gonadotropin-releasing hormone
PV	pituitary gland volume
IPP	idiopathic central precocious puberty
PSM	propensity score matching
